# Supplementary material for: Past political violence and interpersonal violence against children and youth in Africa
Source: Nat Commun. 2026 Apr 13;17:3044. doi: 10.1038/s41467-026-71075-x (PMC13076750; doi:10.1038/s41467-026-71075-x)
Supplement: Supplementary file 1 — Supplementary Information [file 41467_2026_71075_MOESM1_ESM.pdf]

Past political violence and interpersonal violence against children and youth in Africa

22 January 2026

Supplementary Methods

Merging of VACS with ACLED

The VACS have been administered in 25 countries in sub-Saharan Africa, South-East Asia, Latin America and the Caribbean. Several countries have had only one survey round, with second rounds in the pipeline for some other countries.

Supplementary Table 1 summarises data from the VACS for each of the nine selected countries, with survey dates, sample sizes and each country's share of the total sample. Supplementary Table 1 also shows the administrative unit level used to merge the VACS data with conflict data from ACLED. The national survey sample sizes range from 1,819 to 8,715 and cover the period from 2013 to 2019. The total sample size is 35,437 individuals, comprising 11,801 males and 23,636 females.

We merged household-level data from VACS with administrative level aggregated 15-year political violence data from ACLED. For example, for Mozambique we merged the household-level VACS administered in July 2019 with conflict event data that are aggregated at the province level and cover the period from July 2003 to August 2018.

Last 12-month experience of violence data

In three countries, namely Malawi, Nigeria and Zambia, a skip logic was built into questionnaires for the earlier VACS. For each of the experience of violence questions, respondents were first asked whether they had ever experienced this type of violence: if the answer to this question was no, the follow up question about whether this experience occurred during the last 12 months was dropped. For these three countries, we have coded responses to the 12 months question as a no if the answer to the ever question was a no. To validate this approach, we have carefully examined data from the other six countries where there was no such skip and found a consistent pattern in that a no answer to the ever experience question was always followed by a no answer to the last 12 months experience question.

Fatalities measurement

Following the method for measuring the number of political violence, we measure fatalities in each administrative division by aggregating data over 15 years, standardizing the rate per 100,000 population for equitable comparisons, and expressing results in standard deviations to capture variability in political violence within the county.

Supplementary Tables

Table 1. Selected countries with VACS samples and ACLED 15-year political violence data aggregation by lowest common administrative unit/division.

Selected countries	VACS date	VACS sample size	Male	Female	VACS sample share (%)	Administrative unit/division (merge level)	15-Year period of political violence aggregation from ACLED
Côte d'Ivoire	Jun 2018	2,408	1,208	1,200	7%	Region (2)	Jun 2002-Aug 2016
Kenya	Jan 2019	2,130	788	1,342	6%	County (2)	Jan 2003- Dec 2017
Malawi	Sep 2013	2,162	1,133	1,029	6%	District (1)	Sep 1997-Sep 2012
Mozambique	Jul 2019	3,008	879	2,129	8%	Province (2)	Jul 2003-Aug 2018
Namibia	Mar 2019	5,190	980	4,210	15%	Region (2)	Mar 2003-May 2018
Nigeria	May 2014	4,203	2,437	1,766	12%	LGA (1)	May 1998-Jul 2013
Uganda	Sep 2015	5,804	2,645	3,159	16%	District (1)	Sep 1999-Dec 2013
Zambia	Aug 2014	1,819	928	891	5%	District (1)	Aug 1998-Sep 2013
Zimbabwe	Jan 2017	8,715	803	7,912	25%	District (1)	Jan 2001-Aug 2015
**Total**		**35,439**	**11,801**	**23,636**	**100%**		

Data sources: Armed Conflict Location and Event Data (ACLED) and Violence Against Children and Youth Surveys (VACS).

Table 2. Violence against children: Variables and measurements.

	Sexual violence (SV)	Emotional Violence (EV)	Physical Violence (PV)			
			**PV-Partner**	**PV-Family**	**PV-Peers**	**PV-Community**
**Questions**	Touching: Has anyone ever touched you in a sexual way without your permission, but did not try and force you to have sex?	Has a parent, adult caregiver or other adult relative ever:	Has a romantic partner, ex-romantic partner or husband ever:	Has a parent, adult caregiver, or other adult relative ever:	Has a person your own age (not including a girlfriend, wife, romantic partner) ever:	Have any adults such as teachers, police, employers, religious or community leaders, neighbours, or other adults you don't know:
	Attempted sex: Has an intimate partner ever tried to make you have sex against your will but did not succeed? Has anyone else ever tried to make you have sex against your will but did not succeed?	(a) told you that you were not loved, or did not deserve to be loved	(a) slapped, pushed, shoved, shook, or intentionally threw something at you to hurt you? punched, kicked, whipped, or beat you with an object?			
	Physically forced sex: Has anyone else ever physically forced you to have sex and did succeed?	(b) said they wished you had never been born or were dead	(b) choked, smothered, tried to drown you, or burned you intentionally?			
	Pressured sex: Has anyone else ever pressured you to have sex and did succeed?	(c) ridiculed you or put you down, for example said that you were stupid or useless	(c) used or threatened you with a knife, or other weapon?			
**Measure-ments**	Touching, attempted sex, forced or pressured sex by anyone	Emotional violence from family members	Physical violence (aggregated) from IPV, peers, caregivers, and community members			

Table 3. Association between aggregate political violence (PolV) and past year violence against children (VAC) by PolV exposure duration.

	(1)	(2)	(3)	(4)	(5)
**Panel a**	PV	SV	EV	AV	MV
PolV (1 year)	0.993	1.007	1.04	1.003	1.03
P-value	0.841	0.850	0.183	0.932	0.363
Observations	35,325	34,758	34,940	35,418	34,216
Pseudo R-squared	0.0830	0.0611	0.0418	0.0611	0.0555
**Panel b**	PV	SV	EV	AV	MV
PolV (3 years)	0.976	1.006	1.020	0.988	1.017
P-value	0.538	0.860	0.490	0.758	0.627
Observations	35,325	34,758	34,940	35,418	34,216
Pseudo R-squared	0.0831	0.0611	0.0417	0.0611	0.0554
**Panel c**	PV	SV	EV	AV	MV
PolV (5 years)	0.972	1.025	1.031	0.996	1.014
P-value	0.461	0.336	0.178	0.904	0.689
Observations	35,325	34,758	34,940	35,418	34,216
Pseudo R-squared	0.0831	0.0611	0.0418	0.0611	0.0554
**Panel d**	PV	SV	EV	AV	MV
PolV (15 years)	1.013	1.024	1.055	1.030	1.043
P-value	0.386	0.230	<0.001	0.012	0.007
Observations	35,325	34,758	34,940	35,418	34,216
Pseudo R-squared	0.0830	0.0611	0.0421	0.0612	0.0557

Data are presented as adjusted odds ratios (AOR) with *P*-values in parentheses. *P*-values are two-sided and derived from multivariable logistic regression models. Models control for individual and household-level factors, country dummies, and subnational administrative area size (km^2). Sample sizes (n) for each model are listed in the Observations row. PV: Physical Violence; SV: Sexual Violence; EV: Emotional Violence; AV: Any Violence; MV: Multiple Violence.

Table 3a. Logistic regression results of political violence and past year VAC (Physical Violence).

	(1)	(2)	(3)	(4)
	AOR<br>(*P*-value)	AOR<br>(*P*-value)	AOR<br>(*P*-value)	AOR<br>(*P*-value)
Political violence rate (15 years)	1.015<br>(0.390)	1.000<br>(0.989)	1.014<br>(0.378)	1.013<br>(0.385)
Respondent age			0.748<br>(0.001)	0.748<br>(0.001)
Respondent age squared			1.004<br>(0.083)	1.004<br>(0.082)
Gender			0.920<br>(0.227)	0.921<br>(0.231)
Marital status			0.806<br>(0.023)	0.806<br>(0.019)
Child in school			1.680<br>(< 0.001)	1.681<br>(< 0.001)
Respondent worked			1.506<br>(< 0.001)	1.507<br>(< 0.001)
Poorest household				1.014<br>(0.901)
Poorer household				0.995<br>(0.961)
Average household				1.067<br>(0.557)
Richer household				0.988<br>(0.903)
Cote d'Ivoire		3.068<br>(< 0.001)	2.809<br>(< 0.001)	2.803<br>(< 0.001)
Kenya		2.906<br>(< 0.001)	2.545<br>(< 0.001)	2.543<br>(< 0.001)
Malawi		3.966<br>(< 0.001)	4.129<br>(< 0.001)	4.140<br>(< 0.001)
Mozambique		1.965<br>(0.008)	1.984<br>(0.007)	1.985<br>(0.008)
Namibia		2.873<br>(< 0.001)	2.567<br>(< 0.001)	2.566<br>(< 0.001)
Nigeria		2.010<br>(< 0.001)	2.176<br>(< 0.001)	2.173<br>(< 0.001)
Uganda		3.980<br>(< 0.001)	3.642<br>(< 0.001)	3.651<br>(< 0.001)
Zambia		1.957<br>(< 0.001)	1.969<br>(< 0.001)	1.968<br>(< 0.001)
Admin size	0.914<br>(< 0.001)	0.925<br>(0.048)	0.927<br>(0.053)	0.927<br>(0.055)
Constant	0.358<br>(< 0.001)	0.146<br>(< 0.001)	4.382<br>(0.066)	4.336<br>(0.075)
Observations	35,325	35,325	35,325	35,325
Pseudo R-squared	0.00248	0.0187	0.0829	0.0830

Adjusted odds ratios (AOR) with *P*-values in parentheses. *P*-values are two-sided and derived from multivariable logistic regression models. Sample sizes (n) for each model are listed in the Observations row.

Table 3b. Logistic regression results of political violence and past year VAC (Sexual Violence).

	(1)	(2)	(3)	(4)
	AOR (*P*-value)	AOR (*P*-value)	AOR (*P*-value)	AOR (*P*-value)
Political violence rate (15 years)	1.021	1.019	1.026	1.024
	(0.509)	(0.334)	(0.220)	(0.236)
Respondent age			2.261	2.248
			(< 0.001)	(< 0.001)
Respondent age squared			0.980	0.980
			(< 0.001)	(< 0.001)
Gender			2.262	2.233
			(< 0.001)	(< 0.001)
Marital status			0.411	0.426
			(< 0.001)	(< 0.001)
Child in school			1.285	1.252
			(0.003)	(0.006)
Respondent worked			1.142	1.135
			(0.135)	(0.151)
Poorest household				0.805
				(0.066)
Poorer household				0.786
				(0.032)
Average household				0.867
				(0.209)
Richer household				1.081
				(0.433)
Cote d'Ivoire		5.981	5.843	6.062
		(< 0.001)	(< 0.001)	(< 0.001)
Kenya		3.944	3.642	3.675
		(< 0.001)	(< 0.001)	(< 0.001)
Malawi		4.386	5.047	5.096
		(< 0.001)	(< 0.001)	(< 0.001)
Mozambique		5.368	6.356	6.217
		(< 0.001)	(< 0.001)	(< 0.001)
Namibia		4.338	3.692	3.766
		(< 0.001)	(< 0.001)	(< 0.001)
Nigeria		4.905	5.255	5.265
		(< 0.001)	(< 0.001)	(< 0.001)
Uganda		8.388	9.185	9.226
		(< 0.001)	(< 0.001)	(< 0.001)
Zambia		2.667	2.902	2.875
		(< 0.001)	(< 0.001)	(< 0.001)
Admin size	0.860	0.869	0.881	0.882
	(0.001)	(0.009)	(0.012)	(0.011)
Constant	0.150	0.030	0.000	0.000
	(< 0.001)	(< 0.001)	(< 0.001)	(< 0.001)
Observations	34,758	34,758	34,758	34,758
Pseudo R-squared	0.00460	0.0210	0.0591	0.0611

Adjusted odds ratios (AOR) with *P*-values in parentheses. *P*-values are two-sided and derived from multivariable logistic regression models. Sample sizes (n) for each model are listed in the Observations row.

Table 3c. Logistic regression results of political violence and past year VAC (Emotional Violence).

	(1)	(2)	(3)	(4)
	AOR<br>(*P*-value)	AOR<br>(*P*-value)	AOR<br>(*P*-value)	AOR<br>(*P*-value)
Political violence rate (15 years)	1.059<br>(< 0.001)	1.045<br>(0.001)	1.054<br>(< 0.001)	1.055<br>(< 0.001)
Respondent age			1.134<br>(0.363)	1.128<br>(0.381)
Respondent age squared			0.995<br>(0.239)	0.995<br>(0.244)
Gender			1.030<br>(0.744)	1.022<br>(0.805)
Marital status			0.607<br>(< 0.001)	0.617<br>(< 0.001)
Child in school			1.175<br>(0.068)	1.155<br>(0.105)
Respondent worked			1.585<br>(< 0.001)	1.581<br>(< 0.001)
Poorest household				0.808<br>(0.078)
Poorer household				0.947<br>(0.666)
Average household				0.788<br>(0.057)
Richer household				0.954<br>(0.639)
Cote d'Ivoire		1.887<br>(< 0.001)	1.626<br>(< 0.001)	1.647<br>(< 0.001)
Kenya		1.010<br>(0.953)	0.864<br>(0.388)	0.863<br>(0.377)
Malawi		3.219<br>(< 0.001)	3.322<br>(< 0.001)	3.307<br>(< 0.001)
Mozambique		0.742<br>(0.133)	0.724<br>(0.099)	0.711<br>(0.077)
Namibia		1.630<br>(0.013)	1.388<br>(0.098)	1.408<br>(0.078)
Nigeria		1.677<br>(< 0.001)	1.797<br>(< 0.001)	1.796<br>(< 0.001)
Uganda		2.677<br>(< 0.001)	2.285<br>(< 0.001)	2.258<br>(< 0.001)
Zambia		1.827<br>(0.003)	1.851<br>(0.002)	1.852<br>(0.001)
Admin size	0.815<br>(< 0.001)	0.926<br>(0.128)	0.940<br>(0.196)	0.941<br>(0.194)
Constant	0.141<br>(< 0.001)	0.079<br>(< 0.001)	0.034<br>(0.005)	0.041<br>(0.008)
Observations	34,940	34,940	34,940	34,940
Pseudo R-squared	0.00741	0.0230	0.0408	0.0421

Adjusted odds ratios (AOR) with *P*-values in parentheses. *P*-values are two-sided and derived from multivariable logistic regression models. Sample sizes (n) for each model are listed in the Observations row.

Table 3d. Logistic regression results of political violence and past year VAC (Any Violence).

	(1)	(2)	(3)	(4)
	AOR (*P*-value)	AOR (*P*-value)	AOR (*P*-value)	AOR (*P*-value)
Political violence rate (15 years)	1.024	1.018	1.031	1.030
	(0.183)	(0.025)	(0.011)	(0.013)
Respondent age			0.921	0.920
			(0.318)	(0.314)
Respondent age squared			1.000	1.000
			(0.862)	(0.868)
Gender			1.190	1.185
			(0.011)	(0.012)
Marital status			0.598	0.605
			(< 0.001)	(< 0.001)
Child in school			1.480	1.467
			(< 0.001)	(< 0.001)
Respondent worked			1.496	1.494
			(< 0.001)	(< 0.001)
Poorest household				0.946
				(0.550)
Poorer household				0.928
				(0.426)
Average household				0.977
				(0.795)
Richer household				1.016
				(0.844)
Cote d'Ivoire		3.005	2.710	2.718
		(< 0.001)	(< 0.001)	(< 0.001)
Kenya		2.505	2.161	2.159
		(< 0.001)	(< 0.001)	(< 0.001)
Malawi		3.740	4.029	4.030
		(< 0.001)	(< 0.001)	(< 0.001)
Mozambique		2.093	2.198	2.176
		(0.005)	(0.002)	(0.002)
Namibia		2.662	2.290	2.293
		(< 0.001)	(< 0.001)	(< 0.001)
Nigeria		2.289	2.489	2.484
		(< 0.001)	(< 0.001)	(< 0.001)
Uganda		4.638	4.400	4.394
		(< 0.001)	(< 0.001)	(< 0.001)
Zambia		2.004	2.049	2.039
		(< 0.001)	(< 0.001)	(< 0.001)
Admin size	0.880	0.903	0.908	0.908
	(< 0.001)	(0.009)	(0.010)	(0.010)
Constant	0.602	0.233	0.673	0.713
	(< 0.001)	(< 0.001)	(0.596)	(0.653)
Observations	35,418	35,418	35,418	35,418
Pseudo R-squared	0.00525	0.0212	0.0610	0.0612

Adjusted odds ratios (AOR) with *P*-values in parentheses. *P*-values are two-sided and derived from multivariable logistic regression models. Sample sizes (n) for each model are listed in the Observations row.

Table 3e. Logistic regression results of political violence and past year VAC (Multiple Violence).

	(1)	(2)	(3)	(4)
	AOR<br>(*P*-value)	AOR<br>(*P*-value)	AOR<br>(*P*-value)	AOR<br>(*P*-value)
Political violence rate (15 years)	1.059<br>(0.003)	1.031<br>(0.013)	1.043<br>(0.007)	1.043<br>(0.006)
Respondent age			1.367<br>(0.016)	1.360<br>(0.017)
Respondent age squared			0.990<br>(0.006)	0.990<br>(0.006)
Gender			1.299<br>(0.003)	1.289<br>(0.004)
Marital status			0.552<br>(< 0.001)	0.568<br>(< 0.001)
Child in school			1.533<br>(< 0.001)	1.496<br>(< 0.001)
Respondent worked			1.447<br>(< 0.001)	1.443<br>(< 0.001)
Poorest household				0.771<br>(0.050)
Poorer household				0.861<br>(0.232)
Average household				0.854<br>(0.208)
Richer household				0.987<br>(0.914)
Cote d'Ivoire		4.619<br>(< 0.001)	4.109<br>(< 0.001)	4.184<br>(< 0.001)
Kenya		2.590<br>(< 0.001)	2.194<br>(< 0.001)	2.194<br>(< 0.001)
Malawi		5.444<br>(< 0.001)	5.469<br>(< 0.001)	5.472<br>(< 0.001)
Mozambique		2.070<br>(0.004)	2.138<br>(0.001)	2.085<br>(0.001)
Namibia		3.219<br>(< 0.001)	2.696<br>(< 0.001)	2.750<br>(< 0.001)
Nigeria		2.909<br>(< 0.001)	3.108<br>(< 0.001)	3.087<br>(< 0.001)
Uganda		5.844<br>(< 0.001)	5.275<br>(< 0.001)	5.239<br>(< 0.001)
Zambia		2.375<br>(< 0.001)	2.346<br>(< 0.001)	2.331<br>(< 0.001)
Admin size	0.849<br>(0.001)	0.905<br>(0.071)	0.916<br>(0.087)	0.916<br>(0.081)
Constant	0.131<br>(< 0.001)	0.039<br>(< 0.001)	0.003<br>(< 0.001)	0.003<br>(< 0.001)
Observations	34,216	34,216	34,216	34,216
Pseudo R-squared	0.00551	0.0246	0.0544	0.0557

Adjusted odds ratios (AOR) with *P*-values in parentheses. *P*-values are two-sided and derived from multivariable logistic regression models. Sample sizes (n) for each model are listed in the Observations row.

Table 4a. Logistic regression results of political violence (Battle) and past year VAC.

	PV	SV	EV	AV	MV
	AOR (*P*-value)	AOR (*P*-value)	AOR (*P*-value)	AOR (*P*-value)	AOR (*P*-value)
Battle	1.009	1.020	1.038	1.021	1.033
	(0.503)	(0.118)	(< 0.001)	(0.035)	(0.002)
Respondent age	0.748	2.248	1.128	0.920	1.360
	(0.001)	(< 0.001)	(0.380)	(0.314)	(0.017)
Respondent age squared	1.004	0.980	0.995	1.000	0.990
	(0.082)	(< 0.001)	(0.244)	(0.869)	(0.006)
Gender	0.921	2.233	1.022	1.186	1.289
	(0.231)	(< 0.001)	(0.803)	(0.012)	(0.004)
Marital status	0.806	0.426	0.617	0.604	0.567
	(0.019)	(< 0.001)	(< 0.001)	(< 0.001)	(< 0.001)
Child in school	1.681	1.252	1.155	1.467	1.496
	(< 0.001)	(0.006)	(0.105)	(< 0.001)	(< 0.001)
Respondent worked	1.507	1.135	1.581	1.494	1.443
	(< 0.001)	(0.151)	(< 0.001)	(< 0.001)	(< 0.001)
Poorest household	1.014	0.806	0.808	0.945	0.771
	(0.903)	(0.067)	(0.077)	(0.546)	(0.050)
Poorer household	0.994	0.786	0.946	0.928	0.861
	(0.959)	(0.032)	(0.659)	(0.421)	(0.232)
Average household	1.067	0.866	0.787	0.976	0.854
	(0.558)	(0.207)	(0.056)	(0.789)	(0.205)
Richer household	0.988	1.080	0.954	1.015	0.987
	(0.903)	(0.435)	(0.638)	(0.847)	(0.912)
Cote d'Ivoire	2.776	5.899	1.574	2.652	4.015
	(< 0.001)	(< 0.001)	(< 0.001)	(< 0.001)	(< 0.001)
Kenya	2.518	3.599	0.826	2.110	2.118
	(< 0.001)	(< 0.001)	(0.251)	(< 0.001)	(< 0.001)
Malawi	4.095	4.987	3.158	3.933	5.271
	(< 0.001)	(< 0.001)	(< 0.001)	(< 0.001)	(< 0.001)
Mozambique	1.964	6.091	0.680	2.124	2.012
	(0.009)	(< 0.001)	(0.044)	(0.002)	(0.002)
Namibia	2.540	3.691	1.349	2.241	2.656
	(< 0.001)	(< 0.001)	(0.123)	(< 0.001)	(< 0.001)
Nigeria	2.150	5.149	1.714	2.423	2.971
	(< 0.001)	(< 0.001)	(< 0.001)	(< 0.001)	(< 0.001)
Uganda	3.618	9.039	2.168	4.300	5.064
	(< 0.001)	(< 0.001)	(< 0.001)	(< 0.001)	(< 0.001)
Zambia	1.948	2.817	1.772	1.991	2.250
	(< 0.001)	(< 0.001)	(0.003)	(< 0.001)	(< 0.001)
Admin size	0.927	0.882	0.940	0.908	0.915
	(0.055)	(0.011)	(0.192)	(0.010)	(0.080)
Constant	4.367	0.000	0.043	0.725	0.003
	(0.074)	(< 0.001)	(0.009)	(0.670)	(< 0.001)
Observations	35,325	34,758	34,940	35,418	34,216
Pseudo R-squared	0.0830	0.0611	0.0420	0.0612	0.0557

Adjusted odds ratios (AOR) with *P*-values in parentheses. *P*-values are two-sided and derived from multivariable logistic regression models. Sample sizes (n) for each model are listed in the Observations row. PV: Physical Violence; SV: Sexual Violence; EV: Emotional Violence; AV: Any Violence; MV: Multiple Violence.

Table 4b. Logistic regression results of political violence (Explosions) past year VAC.

	PV	SV	EV	AV	MV
	AOR (*P*-value)	AOR (*P*-value)	AOR (*P*-value)	AOR (*P*-value)	AOR (*P*-value)
Explosion rates	0.995	1.013	1.023	1.005	1.023
	(0.838)	(0.386)	(0.005)	(0.794)	(0.073)
Respondent age	0.748	2.247	1.128	0.920	1.360
	(0.001)	(< 0.001)	(0.381)	(0.314)	(0.017)
Respondent age squared	1.004	0.980	0.995	1.000	0.990
	(0.083)	(< 0.001)	(0.244)	(0.870)	(0.006)
Gender	0.921	2.233	1.023	1.186	1.290
	(0.233)	(< 0.001)	(0.798)	(0.012)	(0.004)
Marital status	0.807	0.426	0.618	0.605	0.568
	(0.020)	(< 0.001)	(< 0.001)	(< 0.001)	(< 0.001)
Child in school	1.679	1.252	1.155	1.466	1.495
	(< 0.001)	(0.006)	(0.106)	(< 0.001)	(< 0.001)
Respondent worked	1.507	1.136	1.582	1.495	1.444
	(< 0.001)	(0.150)	(< 0.001)	(< 0.001)	(< 0.001)
Poorest household	1.013	0.807	0.809	0.945	0.773
	(0.907)	(0.068)	(0.081)	(0.545)	(0.052)
Poorer household	0.994	0.787	0.947	0.928	0.862
	(0.955)	(0.033)	(0.668)	(0.421)	(0.237)
Average household	1.068	0.867	0.789	0.977	0.855
	(0.554)	(0.211)	(0.059)	(0.799)	(0.212)
Richer household	0.988	1.081	0.955	1.016	0.988
	(0.904)	(0.429)	(0.651)	(0.839)	(0.923)
Cote d'Ivoire	2.822	5.958	1.608	2.706	4.069
	(< 0.001)	(< 0.001)	(< 0.001)	(< 0.001)	(< 0.001)
Kenya	2.520	3.599	0.826	2.111	2.117
	(< 0.001)	(< 0.001)	(0.249)	(< 0.001)	(< 0.001)
Malawi	4.093	4.986	3.156	3.930	5.270
	(< 0.001)	(< 0.001)	(< 0.001)	(< 0.001)	(< 0.001)
Mozambique	1.957	6.086	0.678	2.118	2.010
	(0.009)	(< 0.001)	(0.042)	(0.002)	(0.002)
Namibia	2.536	3.686	1.344	2.237	2.650
	(< 0.001)	(< 0.001)	(0.126)	(< 0.001)	(< 0.001)
Nigeria	2.151	5.151	1.715	2.424	2.972
	(< 0.001)	(< 0.001)	(< 0.001)	(< 0.001)	(< 0.001)
Uganda	3.627	9.078	2.187	4.321	5.101
	(< 0.001)	(< 0.001)	(< 0.001)	(< 0.001)	(< 0.001)
Zambia	1.947	2.817	1.772	1.991	2.250
	(< 0.001)	(< 0.001)	(0.003)	(< 0.001)	(< 0.001)
Admin size	0.927	0.882	0.941	0.909	0.916
	(0.057)	(0.012)	(0.198)	(0.010)	(0.081)
Constant	4.354	0.000	0.043	0.723	0.003
	(0.075)	(< 0.001)	(0.009)	(0.667)	(< 0.001)
Observations	35,325	34,758	34,940	35,418	34,216
Pseudo R-squared	0.0830	0.0611	0.0418	0.0611	0.0556

Adjusted odds ratios (AOR) with *P*-values in parentheses. *P*-values are two-sided and derived from multivariable logistic regression models. Sample sizes (n) for each model are listed in the Observations row. PV: Physical Violence; SV: Sexual Violence; EV: Emotional Violence; AV: Any Violence; MV: Multiple Violence.

Table 4c. Logistic regression results of political violence (Violence against civilians) and past year VAC.

	PV	SV	EV	AV	MV
	AOR (*P*-value)	AOR (*P*-value)	AOR (*P*-value)	AOR (*P*-value)	AOR (*P*-value)
Violence against civilians rates	1.010	1.044	1.104	1.043	1.072
	(0.782)	(0.357)	(< 0.001)	(0.183)	(0.098)
Respondent age	0.748	2.248	1.128	0.920	1.361
	(0.001)	(< 0.001)	(0.380)	(0.314)	(0.017)
Respondent age squared	1.004	0.980	0.995	1.000	0.990
	(0.082)	(< 0.001)	(0.243)	(0.870)	(0.006)
Gender	0.921	2.234	1.023	1.186	1.290
	(0.232)	(< 0.001)	(0.795)	(0.012)	(0.004)
Marital status	0.806	0.426	0.618	0.605	0.569
	(0.019)	(< 0.001)	(< 0.001)	(< 0.001)	(< 0.001)
Child in school	1.680	1.252	1.155	1.467	1.495
	(< 0.001)	(0.006)	(0.106)	(< 0.001)	(< 0.001)
Respondent worked	1.507	1.136	1.582	1.495	1.444
	(< 0.001)	(0.151)	(< 0.001)	(< 0.001)	(< 0.001)
Poorest household	1.014	0.806	0.811	0.947	0.772
	(0.900)	(0.067)	(0.084)	(0.559)	(0.051)
Poorer household	0.995	0.787	0.951	0.930	0.862
	(0.962)	(0.033)	(0.691)	(0.434)	(0.238)
Average household	1.068	0.868	0.792	0.979	0.857
	(0.553)	(0.213)	(0.063)	(0.812)	(0.217)
Richer household	0.988	1.082	0.956	1.017	0.989
	(0.906)	(0.428)	(0.656)	(0.833)	(0.924)
Cote d'Ivoire	2.831	6.348	1.828	2.832	4.507
	(< 0.001)	(< 0.001)	(< 0.001)	(< 0.001)	(< 0.001)
Kenya	2.550	3.829	0.945	2.228	2.334
	(< 0.001)	(< 0.001)	(0.747)	(< 0.001)	(< 0.001)
Malawi	4.148	5.317	3.637	4.161	5.832
	(< 0.001)	(< 0.001)	(< 0.001)	(< 0.001)	(< 0.001)
Mozambique	1.987	6.479	0.779	2.243	2.215
	(0.009)	(< 0.001)	(0.208)	(0.001)	(0.001)
Namibia	2.571	3.925	1.543	2.366	2.924
	(< 0.001)	(< 0.001)	(0.030)	(< 0.001)	(< 0.001)
Nigeria	2.178	5.491	1.974	2.564	3.288
	(< 0.001)	(< 0.001)	(< 0.001)	(< 0.001)	(< 0.001)
Uganda	3.666	9.608	2.472	4.536	5.573
	(< 0.001)	(< 0.001)	(< 0.001)	(< 0.001)	(< 0.001)
Zambia	1.973	2.998	2.034	2.104	2.482
	(< 0.001)	(< 0.001)	(< 0.001)	(< 0.001)	(< 0.001)
Admin size	0.927	0.882	0.942	0.908	0.916
	(0.056)	(0.011)	(0.198)	(0.010)	(0.082)
Constant	4.321	0.000	0.038	0.695	0.003
	(0.075)	(< 0.001)	(0.007)	(0.629)	(< 0.001)
Observations	35,325	34,758	34,940	35,418	34,216
Pseudo R-squared	0.0830	0.0611	0.0421	0.0612	0.0556

Adjusted odds ratios (AOR) with *P*-values in parentheses. *P*-values are two-sided and derived from multivariable logistic regression models. Sample sizes (n) for each model are listed in the Observations row. PV: Physical Violence; SV: Sexual Violence; EV: Emotional Violence; AV: Any Violence; MV: Multiple Violence.

Table 4d. Logistic regression results of political violence (Riots) and past year VAC.

	PV	SV	EV	AV	MV
	AOR (*P*-value)	AOR (*P*-value)	AOR (*P*-value)	AOR (*P*-value)	AOR (*P*-value)
Riots rates	1.023	1.038	1.041	1.037	1.041
	(0.106)	(0.001)	(< 0.001)	(0.001)	(< 0.001)
Respondent age	0.747	2.246	1.128	0.919	1.359
	(0.001)	(< 0.001)	(0.382)	(0.311)	(0.017)
Respondent age squared	1.004	0.980	0.995	1.000	0.990
	(0.082)	(< 0.001)	(0.245)	(0.863)	(0.006)
Gender	0.920	2.231	1.022	1.184	1.288
	(0.227)	(< 0.001)	(0.809)	(0.013)	(0.004)
Marital status	0.804	0.424	0.617	0.603	0.566
	(0.018)	(< 0.001)	(< 0.001)	(< 0.001)	(< 0.001)
Child in school	1.683	1.254	1.156	1.469	1.497
	(< 0.001)	(0.006)	(0.104)	(< 0.001)	(< 0.001)
Respondent worked	1.507	1.136	1.582	1.494	1.444
	(< 0.001)	(0.151)	(< 0.001)	(< 0.001)	(< 0.001)
Poorest household	1.019	0.813	0.816	0.954	0.779
	(0.862)	(0.080)	(0.093)	(0.614)	(0.060)
Poorer household	0.999	0.792	0.954	0.935	0.868
	(0.994)	(0.039)	(0.707)	(0.472)	(0.260)
Average household	1.069	0.869	0.792	0.980	0.857
	(0.548)	(0.217)	(0.062)	(0.823)	(0.220)
Richer household	0.990	1.085	0.958	1.019	0.992
	(0.922)	(0.412)	(0.674)	(0.808)	(0.944)
Cote d'Ivoire	2.749	5.851	1.595	2.636	4.039
	(< 0.001)	(< 0.001)	(< 0.001)	(< 0.001)	(< 0.001)
Kenya	2.502	3.573	0.818	2.090	2.099
	(< 0.001)	(< 0.001)	(0.225)	(< 0.001)	(< 0.001)
Malawi	4.135	5.085	3.215	3.996	5.372
	(< 0.001)	(< 0.001)	(< 0.001)	(< 0.001)	(< 0.001)
Mozambique	1.976	6.178	0.683	2.143	2.030
	(0.008)	(< 0.001)	(0.046)	(0.002)	(0.002)
Namibia	2.522	3.658	1.327	2.214	2.619
	(< 0.001)	(< 0.001)	(0.139)	(< 0.001)	(< 0.001)
Nigeria	2.173	5.264	1.752	2.467	3.039
	(< 0.001)	(< 0.001)	(< 0.001)	(< 0.001)	(< 0.001)
Uganda	3.653	9.227	2.222	4.378	5.187
	(< 0.001)	(< 0.001)	(< 0.001)	(< 0.001)	(< 0.001)
Zambia	1.950	2.829	1.776	1.996	2.256
	(< 0.001)	(< 0.001)	(0.003)	(< 0.001)	(< 0.001)
Admin size	0.927	0.882	0.943	0.909	0.918
	(0.057)	(0.012)	(0.209)	(0.010)	(0.086)
Constant	4.358	0.000	0.042	0.721	0.003
	(0.075)	(< 0.001)	(0.008)	(0.665)	(< 0.001)
Observations	35,325	34,758	34,940	35,418	34,216
Pseudo R-squared	0.0831	0.0613	0.0420	0.0614	0.0558

Adjusted odds ratios (AOR) with *P*-values in parentheses. *P*-values are two-sided and derived from multivariable logistic regression models. Sample sizes (n) for each model are listed in the Observations row. PV: Physical Violence; SV: Sexual Violence; EV: Emotional Violence; AV: Any Violence; MV: Multiple Violence.

Table 4e. Logistic regression results of political violence (Strategic development) past year VAC.

	PV	SV	EV	AV	MV
	AOR (*P*-value)	AOR (*P*-value)	AOR (*P*-value)	AOR (*P*-value)	AOR (*P*-value)
Strategic development rates	0.994	1.014	1.047	1.014	1.028
	(0.826)	(0.619)	(< 0.001)	(0.543)	(0.276)
Respondent age	0.748	2.247	1.128	0.920	1.360
	(0.001)	(< 0.001)	(0.381)	(0.314)	(0.017)
Respondent age squared	1.004	0.980	0.995	1.000	0.990
	(0.083)	(< 0.001)	(0.244)	(0.870)	(0.006)
Gender	0.921	2.234	1.023	1.186	1.290
	(0.233)	(< 0.001)	(0.798)	(0.012)	(0.004)
Marital status	0.807	0.426	0.618	0.605	0.569
	(0.020)	(< 0.001)	(< 0.001)	(< 0.001)	(< 0.001)
Child in school	1.680	1.251	1.155	1.466	1.495
	(< 0.001)	(0.006)	(0.106)	(< 0.001)	(< 0.001)
Respondent worked	1.507	1.136	1.582	1.494	1.444
	(< 0.001)	(0.150)	(< 0.001)	(< 0.001)	(< 0.001)
Poorest household	1.013	0.806	0.810	0.946	0.772
	(0.906)	(0.067)	(0.081)	(0.549)	(0.051)
Poorer household	0.994	0.787	0.949	0.928	0.862
	(0.954)	(0.033)	(0.677)	(0.425)	(0.236)
Average household	1.068	0.868	0.790	0.977	0.856
	(0.555)	(0.212)	(0.059)	(0.801)	(0.214)
Richer household	0.988	1.082	0.956	1.016	0.988
	(0.903)	(0.428)	(0.654)	(0.837)	(0.924)
Cote d'Ivoire	2.809	6.048	1.638	2.717	4.172
	(< 0.001)	(< 0.001)	(< 0.001)	(< 0.001)	(< 0.001)
Kenya	2.509	3.635	0.852	2.130	2.159
	(< 0.001)	(< 0.001)	(0.335)	(< 0.001)	(< 0.001)
Malawi	4.074	5.038	3.270	3.972	5.382
	(< 0.001)	(< 0.001)	(< 0.001)	(< 0.001)	(< 0.001)
Mozambique	1.950	6.125	0.699	2.137	2.038
	(0.009)	(< 0.001)	(0.062)	(0.002)	(0.002)
Namibia	2.527	3.717	1.386	2.257	2.694
	(< 0.001)	(< 0.001)	(0.091)	(< 0.001)	(< 0.001)
Nigeria	2.141	5.207	1.776	2.449	3.037
	(< 0.001)	(< 0.001)	(< 0.001)	(< 0.001)	(< 0.001)
Uganda	3.618	9.135	2.228	4.344	5.162
	(< 0.001)	(< 0.001)	(< 0.001)	(< 0.001)	(< 0.001)
Zambia	1.939	2.842	1.829	2.009	2.292
	(< 0.001)	(< 0.001)	(0.002)	(< 0.001)	(< 0.001)
Admin size	0.927	0.882	0.941	0.908	0.917
	(0.057)	(0.012)	(0.196)	(0.010)	(0.083)
Constant	4.367	0.000	0.042	0.719	0.003
	(0.074)	(< 0.001)	(0.008)	(0.662)	(< 0.001)
Observations	35,325	34,758	34,940	35,418	34,216
Pseudo R-squared	0.0830	0.0611	0.0420	0.0611	0.0555

Adjusted odds ratios (AOR) with *P*-values in parentheses. *P*-values are two-sided and derived from multivariable logistic regression models. Sample sizes (n) for each model are listed in the Observations row. PV: Physical Violence; SV: Sexual Violence; EV: Emotional Violence; AV: Any Violence; MV: Multiple Violence.

Table 5a. Logistic regression results of political violence and past year VAC (age effect).

	PV	SV	EV	Any	MV
	AOR (*P*-value)	AOR (*P*-value)	AOR (*P*-value)	AOR (*P*-value)	AOR (*P*-value)
Age group*Political violence rate (15 years)	1.092	1.065	1.003	1.051	1.122
	(0.024)	(0.058)	(0.908)	(0.147)	(< 0.001)
Political violence rate (15 years)	0.964	0.982	1.055	0.999	0.973
	(0.286)	(0.635)	(< 0.001)	(0.980)	(0.378)
Age group (18-24=1)	0.750	0.784	0.674	0.772	0.686
	(0.006)	(0.076)	(0.005)	(0.005)	(0.008)
Respondent age	0.824	2.560	1.298	1.025	1.546
	(0.051)	(< 0.001)	(0.065)	(0.790)	(0.001)
Respondent age squared	1.003	0.977	0.993	0.998	0.988
	(0.284)	(< 0.001)	(0.072)	(0.499)	(0.001)
Gender	0.924	2.240	1.027	1.189	1.295
	(0.250)	(< 0.001)	(0.765)	(0.011)	(0.003)
Marital status	0.798	0.424	0.613	0.601	0.560
	(0.014)	(< 0.001)	(< 0.001)	(< 0.001)	(< 0.001)
Child in school	1.664	1.241	1.138	1.454	1.475
	(< 0.001)	(0.009)	(0.147)	(< 0.001)	(< 0.001)
Respondent worked	1.505	1.134	1.579	1.493	1.439
	(< 0.001)	(0.156)	(< 0.001)	(< 0.001)	(< 0.001)
Poorest household	1.019	0.810	0.811	0.949	0.777
	(0.865)	(0.072)	(0.087)	(0.577)	(0.058)
Poorer household	1.000	0.791	0.954	0.933	0.868
	(0.998)	(0.037)	(0.710)	(0.456)	(0.260)
Average household	1.072	0.869	0.791	0.980	0.859
	(0.531)	(0.220)	(0.063)	(0.823)	(0.224)
Richer household	0.992	1.085	0.961	1.019	0.993
	(0.936)	(0.411)	(0.693)	(0.806)	(0.956)
Cote d'Ivoire	2.779	6.052	1.652	2.713	4.136
	(< 0.001)	(< 0.001)	(< 0.001)	(< 0.001)	(< 0.001)
Kenya	2.534	3.685	0.868	2.162	2.186
	(< 0.001)	(< 0.001)	(0.399)	(< 0.001)	(< 0.001)
Malawi	4.140	5.106	3.348	4.052	5.456
	(< 0.001)	(< 0.001)	(< 0.001)	(< 0.001)	(< 0.001)
Mozambique	1.974	6.231	0.715	2.178	2.070
	(0.009)	(< 0.001)	(0.081)	(0.002)	(0.002)
Namibia	2.568	3.795	1.427	2.308	2.753
	(< 0.001)	(< 0.001)	(0.068)	(< 0.001)	(< 0.001)
Nigeria	2.159	5.284	1.805	2.485	3.065
	(< 0.001)	(< 0.001)	(< 0.001)	(< 0.001)	(< 0.001)
Uganda	3.643	9.260	2.265	4.402	5.237
	(< 0.001)	(< 0.001)	(< 0.001)	(< 0.001)	(< 0.001)
Zambia	1.952	2.880	1.860	2.036	2.310
	(< 0.001)	(< 0.001)	(0.001)	(< 0.001)	(< 0.001)
Admin size	0.926	0.881	0.940	0.907	0.915
	(0.054)	(0.011)	(0.189)	(0.009)	(0.079)
Constant	1.508	0.000	0.009	0.231	0.001
	(0.656)	(< 0.001)	(< 0.001)	(0.097)	(< 0.001)
Observations	35,325	34,758	34,940	35,418	34,216
Pseudo R-squared	0.0841	0.0618	0.0434	0.0620	0.0576

Adjusted odds ratios (AOR) with *P*-values in parentheses. *P*-values are two-sided and derived from multivariable logistic regression models. Sample sizes (n) for each model are listed in the Observations row. PV: Physical Violence; SV: Sexual Violence; EV: Emotional Violence; AV: Any Violence; MV: Multiple Violence.

Table 5b. Logistic regression results of political violence and past year VAC (gender effect).

	PV	SV	EV	Any	MV
	AOR (*P*-value)	AOR (*P*-value)	AOR (*P*-value)	AOR (*P*-value)	AOR (*P*-value)
Gender*Political violence rate (15 years)	1.031 (0.118)	0.972 (0.398)	1.027 (0.565)	1.021 (0.513)	1.010 (0.745)
Political violence rate (15 years)	0.994 (0.805)	1.044 (0.272)	1.037 (0.146)	1.016 (0.594)	1.036 (0.236)
Gender (Female=1)	0.927 (0.256)	2.221 (< 0.001)	1.027 (0.765)	1.190 (0.009)	1.291 (0.003)
Respondent age	0.748 (0.001)	2.248 (< 0.001)	1.128 (0.380)	0.920 (0.314)	1.360 (0.017)
Respondent age squared	1.004 (0.082)	0.980 (< 0.001)	0.995 (0.244)	1.000 (0.869)	0.990 (0.006)
Marital status	0.805 (0.019)	0.426 (< 0.001)	0.617 (< 0.001)	0.604 (< 0.001)	0.568 (< 0.001)
Child in school	1.681 (< 0.001)	1.252 (0.006)	1.155 (0.105)	1.467 (< 0.001)	1.496 (< 0.001)
Respondent worked	1.507 (< 0.001)	1.135 (0.153)	1.582 (< 0.001)	1.495 (< 0.001)	1.444 (< 0.001)
Poorest household	1.014 (0.900)	0.805 (0.066)	0.808 (0.078)	0.946 (0.550)	0.771 (0.050)
Poorer household	0.995 (0.963)	0.786 (0.032)	0.947 (0.669)	0.928 (0.427)	0.861 (0.233)
Average household	1.067 (0.560)	0.867 (0.210)	0.788 (0.057)	0.976 (0.792)	0.854 (0.207)
Richer household	0.987 (0.901)	1.081 (0.432)	0.954 (0.637)	1.015 (0.846)	0.987 (0.913)
Cote d'Ivoire	2.795 (< 0.001)	6.066 (< 0.001)	1.643 (< 0.001)	2.714 (< 0.001)	4.181 (< 0.001)
Kenya	2.538 (< 0.001)	3.675 (< 0.001)	0.861 (0.370)	2.157 (< 0.001)	2.193 (< 0.001)
Malawi	4.129 (< 0.001)	5.098 (< 0.001)	3.300 (< 0.001)	4.024 (< 0.001)	5.468 (< 0.001)
Mozambique	1.980 (0.008)	6.218 (< 0.001)	0.709 (0.074)	2.172 (0.002)	2.084 (0.001)
Namibia	2.559 (< 0.001)	3.767 (< 0.001)	1.405 (0.081)	2.290 (< 0.001)	2.748 (< 0.001)
Nigeria	2.168 (< 0.001)	5.266 (< 0.001)	1.793 (< 0.001)	2.481 (< 0.001)	3.085 (< 0.001)
Uganda	3.647 (< 0.001)	9.221 (< 0.001)	2.256 (< 0.001)	4.392 (< 0.001)	5.238 (< 0.001)
Zambia	1.964 (< 0.001)	2.876 (< 0.001)	1.849 (0.002)	2.036 (< 0.001)	2.330 (< 0.001)
Admin size	0.927 (0.055)	0.882 (0.011)	0.941 (0.195)	0.908 (0.010)	0.916 (0.081)
Constant	4.324 (0.076)	0.000 (< 0.001)	0.041 (0.008)	0.711 (0.651)	0.003 (< 0.001)
Observations	35,325	34,758	34,940	35,418	34,216
Pseudo R-squared	0.0830	0.0612	0.0421	0.0612	0.0557

Adjusted odds ratios (AOR) with *P*-values in parentheses. *P*-values are two-sided and derived from multivariable logistic regression models. Sample sizes (n) for each model are listed in the Observations row. PV: Physical Violence; SV: Sexual Violence; EV: Emotional Violence; AV: Any Violence; MV: Multiple Violence.

Table 5c. Logistic regression results of political violence and past year VAC (wealth effect).

	PV	SV	EV	Any	MV
	AOR (*P*-value)	AOR (*P*-value)	AOR (*P*-value)	AOR (*P*-value)	AOR (*P*-value)
Poor*Political violence rate (15 years)	0.998	1.169	0.958	0.978	1.101
	(0.920)	(< 0.001)	(0.052)	(0.201)	(< 0.001)
Political violence rate (15 years)	1.015	0.915	1.081	1.044	0.975
	(0.549)	(0.012)	(< 0.001)	(0.028)	(0.337)
Wealth (Poor=1)	1.031	0.814	0.862	0.939	0.847
	(0.660)	(0.004)	(0.074)	(0.307)	(0.022)
Respondent age	0.748	2.250	1.130	0.920	1.363
	(0.001)	(< 0.001)	(0.374)	(0.314)	(0.017)
Respondent age squared	1.004	0.980	0.995	1.000	0.990
	(0.081)	(< 0.001)	(0.238)	(0.866)	(0.006)
Gender	0.921	2.231	1.022	1.186	1.288
	(0.234)	(< 0.001)	(0.809)	(0.012)	(0.004)
Marital status	0.803	0.428	0.618	0.603	0.570
	(0.018)	(< 0.001)	(< 0.001)	(< 0.001)	(< 0.001)
Child in school	1.686	1.251	1.154	1.470	1.500
	(< 0.001)	(0.007)	(0.111)	(< 0.001)	(< 0.001)
Respondent worked	1.507	1.137	1.581	1.495	1.444
	(< 0.001)	(0.148)	(< 0.001)	(< 0.001)	(< 0.001)
Cote d'Ivoire	2.807	5.706	1.656	2.733	4.040
	(< 0.001)	(< 0.001)	(< 0.001)	(< 0.001)	(< 0.001)
Kenya	2.548	3.468	0.873	2.169	2.135
	(< 0.001)	(< 0.001)	(0.420)	(< 0.001)	(< 0.001)
Malawi	4.135	4.782	3.354	4.046	5.294
	(< 0.001)	(< 0.001)	(< 0.001)	(< 0.001)	(< 0.001)
Mozambique	1.995	5.836	0.715	2.189	2.020
	(0.008)	(< 0.001)	(0.083)	(0.002)	(0.002)
Namibia	2.567	3.566	1.405	2.304	2.645
	(< 0.001)	(< 0.001)	(0.080)	(< 0.001)	(< 0.001)
Nigeria	2.179	4.969	1.816	2.500	3.003
	(< 0.001)	(< 0.001)	(< 0.001)	(< 0.001)	(< 0.001)
Uganda	3.650	8.617	2.308	4.413	5.077
	(< 0.001)	(< 0.001)	(< 0.001)	(< 0.001)	(< 0.001)
Zambia	1.974	2.717	1.859	2.052	2.255
	(< 0.001)	(< 0.001)	(0.001)	(< 0.001)	(< 0.001)
Admin size	0.926	0.881	0.942	0.908	0.916
	(0.055)	(0.011)	(0.203)	(0.009)	(0.080)
Constant	4.258	0.000	0.040	0.712	0.003
	(0.075)	(< 0.001)	(0.007)	(0.652)	(< 0.001)
Observations	35,325	34,758	34,940	35,418	34,216
Pseudo R-squared	0.0829	0.0615	0.0416	0.0612	0.0558

Adjusted odds ratios (AOR) with *P*-values in parentheses. *P*-values are two-sided and derived from multivariable logistic regression models. Sample sizes (n) for each model are listed in the Observations row. PV: Physical Violence; SV: Sexual Violence; EV: Emotional Violence; AV: Any Violence; MV: Multiple Violence.

Table 6a. Logistic regression results of political violence and past year PV by perpetrators (overall effect).

	PV-Family	PV-Partner	PV-Peer	PV-Community
	AOR (*P*-value)	AOR (*P*-value)	AOR (*P*-value)	AOR (*P*-value)
Political violence rate (15 years)	0.975	1.030	0.999	1.007
	(0.352)	(0.047)	(0.917)	(0.855)
Respondent age	0.875	0.723	0.834	1.318
	(0.347)	(0.320)	(0.122)	(0.141)
Respondent age squared	0.998	1.010	1.002	0.988
	(0.594)	(0.233)	(0.608)	(0.024)
Gender	1.069	2.054	0.493	0.978
	(0.430)	(< 0.001)	(< 0.001)	(0.825)
Marital status	0.356	1.927	0.535	0.302
	(< 0.001)	(< 0.001)	(< 0.001)	(< 0.001)
Child in school	1.566	0.613	1.287	3.245
	(< 0.001)	(0.009)	(0.005)	(< 0.001)
Respondent worked	1.309	1.073	1.539	1.467
	(0.017)	(0.537)	(< 0.001)	(< 0.001)
Poorest household	0.949	1.173	0.975	1.336
	(0.713)	(0.383)	(0.841)	(0.062)
Poorer household	0.922	1.196	0.911	1.233
	(0.543)	(0.302)	(0.445)	(0.152)
Average household	0.920	1.045	0.960	1.341
	(0.540)	(0.817)	(0.762)	(0.048)
Richer household	1.077	1.079	0.829	1.253
	(0.621)	(0.655)	(0.127)	(0.091)
Cote d'Ivoire	3.295	3.973	2.742	2.354
	(< 0.001)	(< 0.001)	(< 0.001)	(< 0.001)
Kenya	3.190	1.699	1.878	5.046
	(< 0.001)	(0.009)	(< 0.001)	(< 0.001)
Malawi	4.276	1.783	3.746	5.909
	(< 0.001)	(0.001)	(< 0.001)	(< 0.001)
Mozambique	1.927	1.962	1.712	1.909
	(0.037)	(0.002)	(0.088)	(0.023)
Namibia	2.626	2.913	2.549	3.769
	(< 0.001)	(< 0.001)	(< 0.001)	(< 0.001)
Nigeria	4.298	0.738	1.589	4.707
	(< 0.001)	(0.093)	(< 0.001)	(< 0.001)
Uganda	3.228	2.692	2.202	7.194
	(< 0.001)	(< 0.001)	(< 0.001)	(< 0.001)
Zambia	2.116	2.165	1.175	2.543
	(< 0.001)	(< 0.001)	(0.310)	(< 0.001)
Admin size	0.935	0.991	0.988	0.792
	(0.178)	(0.756)	(0.790)	(0.014)
Constant	0.540	0.219	1.111	0.005
	(0.615)	(0.646)	(0.921)	(0.001)
Observations	34,978	21,495	34,738	34,976
Pseudo R-squared	0.122	0.0972	0.0847	0.171

Adjusted odds ratios (AOR) with *P*-values in parentheses. *P*-values are two-sided and derived from multivariable logistic regression models. Sample sizes (n) for each model are listed in the Observations row. PV: Physical Violence; PV-family: PV from family members; PV-partner: PV from partner; PV-peer: PV from peer; PV-community: PV from adult in the community.

Table 6b. Logistic regression results of political violence and past year PV by perpetrators (age effect).

	PV-Family	PV-Partner	PV-Peer	PV-Community
	AOR (*P*-value)	AOR (*P*-value)	AOR (*P*-value)	AOR (*P*-value)
Age group*Political violence rate (15 years)	1.157	0.938	0.987	1.047
	(< 0.001)	(0.031)	(0.625)	(0.309)
Political violence rate (15 years)	0.914	1.086	1.005	0.992
	(0.014)	(< 0.001)	(0.813)	(0.880)
Age group (18-24=1)	0.885	0.781	0.755	0.751
	(0.430)	(0.389)	(0.049)	(0.033)
Respondent age	0.885	0.883	0.893	1.355
	(0.384)	(0.808)	(0.343)	(0.093)
Respondent age squared	0.998	1.005	1.001	0.988
	(0.639)	(0.674)	(0.808)	(0.024)
Gender	1.071	2.056	0.494	0.979
	(0.422)	(< 0.001)	(< 0.001)	(0.835)
Marital status	0.352	1.922	0.532	0.298
	(< 0.001)	(< 0.001)	(< 0.001)	(< 0.001)
Child in school	1.560	0.611	1.275	3.221
	(< 0.001)	(0.008)	(0.007)	(< 0.001)
Respondent worked	1.304	1.072	1.540	1.466
	(0.019)	(0.545)	(< 0.001)	(< 0.001)
Poorest household	0.954	1.176	0.976	1.342
	(0.737)	(0.373)	(0.845)	(0.057)
Poorer household	0.926	1.201	0.914	1.242
	(0.561)	(0.289)	(0.464)	(0.139)
Average household	0.921	1.050	0.964	1.345
	(0.544)	(0.801)	(0.785)	(0.045)
Richer household	1.080	1.083	0.831	1.258
	(0.605)	(0.636)	(0.132)	(0.083)
Cote d'Ivoire	3.232	3.958	2.751	2.356
	(< 0.001)	(< 0.001)	(< 0.001)	(< 0.001)
Kenya	3.149	1.700	1.889	5.077
	(< 0.001)	(0.009)	(< 0.001)	(< 0.001)
Malawi	4.211	1.787	3.785	5.949
	(< 0.001)	(0.001)	(< 0.001)	(< 0.001)
Mozambique	1.892	1.966	1.720	1.915
	(0.043)	(0.002)	(0.087)	(0.023)
Namibia	2.592	2.912	2.575	3.810
	(< 0.001)	(< 0.001)	(< 0.001)	(< 0.001)
Nigeria	4.232	0.737	1.595	4.728
	(< 0.001)	(0.090)	(< 0.001)	(< 0.001)
Uganda	3.204	2.691	2.203	7.220
	(< 0.001)	(< 0.001)	(< 0.001)	(< 0.001)
Zambia	2.081	2.158	1.179	2.548
	(< 0.001)	(< 0.001)	(0.302)	(< 0.001)
Admin size	0.935	0.990	0.988	0.791
	(0.178)	(0.752)	(0.783)	(0.014)
Constant	0.436	0.031	0.511	0.003
	(0.497)	(0.500)	(0.543)	(< 0.001)
Observations	34,978	21,495	34,738	34,976
Pseudo R-squared	0.123	0.0976	0.0853	0.172

Adjusted odds ratios (AOR) with *P*-values in parentheses. *P*-values are two-sided and derived from multivariable logistic regression models. Sample sizes (n) for each model are listed in the Observations row. PV: Physical Violence; PV-family: PV from family members; PV-partner: PV from partner; PV-peer: PV from peer; PV-community: PV from adult in the community.

Table 6c. Logistic regression results of political violence and past year PV by perpetrators (gender effect).

	PV-Family	PV-Partner	PV-Peer	PV-Community
	AOR (*P*-value)	AOR (*P*-value)	AOR (*P*-value)	AOR (*P*-value)
Gender*Political violence rate (15 years)	1.042	0.993	1.038	0.944
	(0.238)	(0.752)	(0.155)	(0.421)
Political violence rate (15 years)	0.950	1.035	0.980	1.035
	(0.199)	(0.065)	(0.344)	(0.142)
Gender (Female=1)	1.080	2.053	0.496	0.965
	(0.352)	(< 0.001)	(< 0.001)	(0.722)
Respondent age	0.875	0.723	0.835	1.317
	(0.348)	(0.319)	(0.123)	(0.141)
Respondent age squared	0.998	1.010	1.002	0.988
	(0.593)	(0.232)	(0.609)	(0.024)
Marital status	0.356	1.927	0.535	0.302
	(< 0.001)	(< 0.001)	(< 0.001)	(< 0.001)
Child in school	1.566	0.613	1.287	3.245
	(< 0.001)	(0.009)	(0.005)	(< 0.001)
Respondent worked	1.309	1.073	1.539	1.467
	(0.017)	(0.539)	(< 0.001)	(< 0.001)
Poorest household	0.949	1.173	0.975	1.336
	(0.713)	(0.383)	(0.842)	(0.062)
Poorer household	0.922	1.196	0.911	1.233
	(0.543)	(0.303)	(0.447)	(0.153)
Average household	0.920	1.046	0.960	1.341
	(0.538)	(0.817)	(0.758)	(0.047)
Richer household	1.077	1.079	0.828	1.253
	(0.622)	(0.653)	(0.126)	(0.090)
Cote d'Ivoire	3.284	3.973	2.731	2.355
	(< 0.001)	(< 0.001)	(< 0.001)	(< 0.001)
Kenya	3.182	1.699	1.872	5.044
	(< 0.001)	(0.009)	(< 0.001)	(< 0.001)
Malawi	4.262	1.783	3.732	5.909
	(< 0.001)	(0.001)	(< 0.001)	(< 0.001)
Mozambique	1.921	1.963	1.705	1.909
	(0.038)	(0.002)	(0.091)	(0.023)
Namibia	2.618	2.914	2.539	3.769
	(< 0.001)	(< 0.001)	(< 0.001)	(< 0.001)
Nigeria	4.285	0.738	1.583	4.705
	(< 0.001)	(0.093)	(< 0.001)	(< 0.001)
Uganda	3.224	2.692	2.197	7.186
	(< 0.001)	(< 0.001)	(< 0.001)	(< 0.001)
Zambia	2.110	2.165	1.171	2.542
	(< 0.001)	(< 0.001)	(0.321)	(< 0.001)
Admin size	0.935	0.991	0.988	0.792
	(0.178)	(0.756)	(0.790)	(0.014)
Constant	0.537	0.220	1.108	0.005
	(0.612)	(0.646)	(0.923)	(0.001)
Observations	34,978	21,495	34,738	34,976
Pseudo R-squared	0.122	0.0972	0.0847	0.172

Adjusted odds ratios (AOR) with *P*-values in parentheses. *P*-values are two-sided and derived from multivariable logistic regression models. Sample sizes (n) for each model are listed in the Observations row. PV: Physical Violence; PV-family: PV from family members; PV-partner: PV from partner; PV-peer: PV from peer; PV-community: PV from adult in the community.

Table 6d. Logistic regression results of political violence and past year PV by perpetrators (wealth effect).

	PV-Family	PV-Partner	PV-Peer	PV-Community
	AOR (*P*-value)	AOR (*P*-value)	AOR (*P*-value)	AOR (*P*-value)
Poor*Political violence rate (15 years)	1.009	1.035	1.035	0.965
	(0.793)	(0.021)	(0.404)	(0.240)
Political violence rate (15 years)	0.969	1.010	0.975	1.033
	(0.484)	(0.565)	(0.497)	(0.464)
Wealth (Poor=1)	0.899	1.095	1.042	1.157
	(0.158)	(0.396)	(0.602)	(0.138)
Respondent age	0.873	0.721	0.836	1.313
	(0.340)	(0.309)	(0.126)	(0.144)
Respondent age squared	0.998	1.010	1.002	0.988
	(0.600)	(0.225)	(0.615)	(0.024)
Gender	1.070	2.049	0.492	0.979
	(0.430)	(< 0.001)	(< 0.001)	(0.835)
Marital status	0.357	1.947	0.534	0.303
	(< 0.001)	(< 0.001)	(< 0.001)	(< 0.001)
Child in school	1.560	0.609	1.295	3.235
	(< 0.001)	(0.008)	(0.004)	(< 0.001)
Respondent worked	1.311	1.072	1.532	1.474
	(0.017)	(0.545)	(< 0.001)	(< 0.001)
Cote d'Ivoire	3.285	3.974	2.737	2.370
	(< 0.001)	(< 0.001)	(< 0.001)	(< 0.001)
Kenya	3.171	1.689	1.880	5.040
	(< 0.001)	(0.010)	(< 0.001)	(< 0.001)
Malawi	4.266	1.800	3.709	5.955
	(< 0.001)	(0.001)	(< 0.001)	(< 0.001)
Mozambique	1.910	1.949	1.728	1.893
	(0.039)	(0.002)	(0.090)	(0.025)
Namibia	2.627	2.931	2.545	3.815
	(< 0.001)	(< 0.001)	(< 0.001)	(< 0.001)
Nigeria	4.278	0.737	1.581	4.740
	(< 0.001)	(0.090)	(< 0.001)	(< 0.001)
Uganda	3.206	2.681	2.200	7.157
	(< 0.001)	(< 0.001)	(< 0.001)	(< 0.001)
Zambia	2.108	2.156	1.174	2.559
	(< 0.001)	(< 0.001)	(0.315)	(< 0.001)
Admin size	0.935	0.990	0.987	0.793
	(0.177)	(0.743)	(0.772)	(0.014)
Constant	0.575	0.240	0.996	0.006
	(0.649)	(0.663)	(0.997)	(0.001)
Observations	34,978	21,495	34,738	34,976
Pseudo R-squared	0.122	0.0969	0.0842	0.171

Adjusted odds ratios (AOR) with *P*-values in parentheses. *P*-values are two-sided and derived from multivariable logistic regression models. Sample sizes (n) for each model are listed in the Observations row. PV: Physical Violence; PV-family: PV from family members; PV-partner: PV from partner; PV-peer: PV from peer; PV-community: PV from adult in the community.

Table 7. Logistic regression results of peaceful protest and past year VAC.

	PV	SV	EV	AV	MV
	AOR (*P*-value)	AOR (*P*-value)	AOR (*P*-value)	AOR (*P*-value)	AOR (*P*-value)
Protest rates	1.010	1.172	1.117	1.104	1.039
	(0.922)	(0.231)	(0.280)	(0.274)	(0.750)
Respondent age	0.748	2.247	1.129	0.920	1.361
	(0.001)	(< 0.001)	(0.378)	(0.312)	(0.017)
Respondent age squared	1.004	0.980	0.995	1.000	0.990
	(0.083)	(< 0.001)	(0.241)	(0.869)	(0.006)
Gender	0.921	2.232	1.023	1.185	1.290
	(0.232)	(< 0.001)	(0.800)	(0.012)	(0.004)
Marital status	0.807	0.428	0.622	0.607	0.571
	(0.020)	(< 0.001)	(< 0.001)	(< 0.001)	(< 0.001)
Child in school	1.680	1.254	1.155	1.468	1.493
	(< 0.001)	(0.006)	(0.107)	(< 0.001)	(< 0.001)
Respondent worked	1.507	1.137	1.584	1.495	1.445
	(< 0.001)	(0.146)	(< 0.001)	(< 0.001)	(< 0.001)
Poorest household	1.015	0.825	0.824	0.961	0.776
	(0.890)	(0.106)	(0.123)	(0.676)	(0.060)
Poorer household	0.996	0.804	0.963	0.943	0.865
	(0.969)	(0.053)	(0.773)	(0.533)	(0.256)
Average household	1.069	0.883	0.802	0.990	0.860
	(0.545)	(0.281)	(0.088)	(0.913)	(0.240)
Richer household	0.989	1.097	0.965	1.025	0.992
	(0.911)	(0.349)	(0.727)	(0.756)	(0.945)
Cote d'Ivoire	2.811	6.467	1.713	2.795	4.243
	(< 0.001)	(< 0.001)	(< 0.001)	(< 0.001)	(< 0.001)
Kenya	2.525	3.860	0.857	2.173	2.148
	(< 0.001)	(< 0.001)	(0.372)	(< 0.001)	(< 0.001)
Malawi	4.113	5.547	3.352	4.136	5.381
	(< 0.001)	(< 0.001)	(< 0.001)	(< 0.001)	(< 0.001)
Mozambique	1.968	6.696	0.706	2.213	2.028
	(0.009)	(< 0.001)	(0.085)	(0.001)	(0.002)
Namibia	2.511	3.078	1.175	2.000	2.527
	(< 0.001)	(< 0.001)	(0.481)	(< 0.001)	(0.001)
Nigeria	2.160	5.742	1.827	2.554	3.042
	(< 0.001)	(< 0.001)	(< 0.001)	(< 0.001)	(< 0.001)
Uganda	3.641	10.010	2.315	4.527	5.209
	(< 0.001)	(< 0.001)	(< 0.001)	(< 0.001)	(< 0.001)
Zambia	1.953	3.035	1.844	2.059	2.280
	(< 0.001)	(< 0.001)	(0.003)	(< 0.001)	(< 0.001)
Admin size	0.927	0.885	0.947	0.911	0.919
	(0.057)	(0.014)	(0.232)	(0.011)	(0.089)
Constant	4.355	0.000	0.041	0.712	0.003
	(0.074)	(< 0.001)	(0.008)	(0.652)	(< 0.001)
Observations	35,325	34,758	34,940	35,418	34,216
Pseudo R-squared	0.0830	0.0613	0.0418	0.0612	0.0554

Adjusted odds ratios (AOR) with *P*-values in parentheses. *P*-values are two-sided and derived from multivariable logistic regression models. Sample sizes (n) for each model are listed in the Observations row. PV: Physical Violence; SV: Sexual Violence; EV: Emotional Violence; AV: Any Violence; MV: Multiple Violence.

Table 8a. Logistic regression results of political violence (fatality) and past year VAC.

	PV	SV	EV	AV	MV
	AOR (*P*-value)	AOR (*P*-value)	AOR (*P*-value)	AOR (*P*-value)	AOR (*P*-value)
Fatality rate (15 years)	1.008 (0.477)	1.023 (0.021)	1.033 (< 0.001)	1.018 (0.107)	1.035 (< 0.001)
Respondent age	0.748 (0.001)	2.248 (< 0.001)	1.128 (0.381)	0.920 (0.313)	1.360 (0.017)
Respondent age squared	1.004 (0.082)	0.980 (< 0.001)	0.995 (0.245)	1.000 (0.868)	0.990 (0.006)
Gender	0.921 (0.232)	2.233 (< 0.001)	1.023 (0.798)	1.186 (0.012)	1.289 (0.004)
Marital status	0.805 (0.019)	0.425 (< 0.001)	0.617 (< 0.001)	0.604 (< 0.001)	0.566 (< 0.001)
Child in school	1.681 (< 0.001)	1.252 (0.006)	1.155 (0.105)	1.467 (< 0.001)	1.496 (< 0.001)
Respondent worked	1.507 (< 0.001)	1.136 (0.150)	1.582 (< 0.001)	1.494 (< 0.001)	1.444 (< 0.001)
Poorest household	1.014 (0.902)	0.806 (0.068)	0.808 (0.078)	0.945 (0.546)	0.771 (0.051)
Poorer household	0.994 (0.959)	0.787 (0.033)	0.946 (0.662)	0.928 (0.422)	0.861 (0.234)
Average household	1.067 (0.559)	0.866 (0.207)	0.788 (0.057)	0.976 (0.790)	0.853 (0.204)
Richer household	0.988 (0.902)	1.080 (0.436)	0.954 (0.639)	1.015 (0.847)	0.986 (0.910)
Cote d'Ivoire	2.776 (< 0.001)	5.872 (< 0.001)	1.583 (< 0.001)	2.662 (< 0.001)	3.995 (< 0.001)
Kenya	2.518 (< 0.001)	3.599 (< 0.001)	0.826 (0.252)	2.111 (< 0.001)	2.118 (< 0.001)
Malawi	4.095 (< 0.001)	4.985 (< 0.001)	3.156 (< 0.001)	3.931 (< 0.001)	5.269 (< 0.001)
Mozambique	1.964 (0.009)	6.094 (< 0.001)	0.678 (0.043)	2.122 (0.002)	2.013 (0.002)
Namibia	2.540 (< 0.001)	3.690 (< 0.001)	1.347 (0.124)	2.240 (< 0.001)	2.656 (< 0.001)
Nigeria	2.149 (< 0.001)	5.144 (< 0.001)	1.712 (< 0.001)	2.421 (< 0.001)	2.966 (< 0.001)
Uganda	3.620 (< 0.001)	9.045 (< 0.001)	2.175 (< 0.001)	4.307 (< 0.001)	5.072 (< 0.001)
Zambia	1.948 (< 0.001)	2.815 (< 0.001)	1.771 (0.003)	1.991 (< 0.001)	2.248 (< 0.001)
Admin size	0.927 (0.055)	0.881 (0.011)	0.941 (0.195)	0.908 (0.010)	0.915 (0.080)
Constant	4.369 (0.074)	0.000 (< 0.001)	0.043 (0.009)	0.726 (0.671)	0.003 (< 0.001)
Observations	35,325	34,758	34,940	35,418	34,216
Pseudo R-squared	0.0830	0.0612	0.0419	0.0612	0.0558

Adjusted odds ratios (AOR) with *P*-values in parentheses. *P*-values are two-sided and derived from multivariable logistic regression models. Sample sizes (n) for each model are listed in the Observations row. PV: Physical Violence; SV: Sexual Violence; EV: Emotional Violence; AV: Any Violence; MV: Multiple Violence.

Table 8b. Logistic regression results of political violence (fatality) and past year PV by perpetrators.

	PV-family	PV-partner	PV-peer	PV-community
	AOR (*P*-value)	AOR (*P*-value)	AOR (*P*-value)	AOR (*P*-value)
Fatality rate (15 years)	0.987	1.016	0.999	1.009
	(0.444)	(0.078)	(0.924)	(0.742)
Respondent age	0.875	0.724	0.834	1.318
	(0.348)	(0.320)	(0.122)	(0.141)
Respondent age squared	0.998	1.010	1.002	0.988
	(0.594)	(0.233)	(0.608)	(0.024)
Gender	1.069	2.054	0.493	0.978
	(0.433)	(< 0.001)	(< 0.001)	(0.826)
Marital status	0.356	1.928	0.535	0.302
	(< 0.001)	(< 0.001)	(< 0.001)	(< 0.001)
Child in school	1.566	0.613	1.287	3.246
	(< 0.001)	(0.009)	(0.005)	(< 0.001)
Respondent worked	1.308	1.073	1.539	1.467
	(0.018)	(0.537)	(< 0.001)	(< 0.001)
Poorest household	0.949	1.171	0.975	1.336
	(0.713)	(0.386)	(0.841)	(0.062)
Poorer household	0.922	1.195	0.911	1.233
	(0.545)	(0.304)	(0.446)	(0.152)
Average household	0.921	1.045	0.960	1.341
	(0.541)	(0.817)	(0.762)	(0.048)
Richer household	1.077	1.080	0.829	1.252
	(0.619)	(0.650)	(0.127)	(0.091)
Cote d'Ivoire	3.348	3.911	2.745	2.331
	(< 0.001)	(< 0.001)	(< 0.001)	(< 0.001)
Kenya	3.250	1.661	1.880	5.019
	(< 0.001)	(0.013)	(< 0.001)	(< 0.001)
Malawi	4.365	1.738	3.751	5.876
	(< 0.001)	(0.002)	(< 0.001)	(< 0.001)
Mozambique	1.968	1.911	1.714	1.900
	(0.030)	(0.003)	(0.087)	(0.023)
Namibia	2.679	2.844	2.552	3.752
	(< 0.001)	(< 0.001)	(< 0.001)	(< 0.001)
Nigeria	4.389	0.719	1.591	4.677
	(< 0.001)	(0.070)	(< 0.001)	(< 0.001)
Uganda	3.278	2.636	2.204	7.154
	(< 0.001)	(< 0.001)	(< 0.001)	(< 0.001)
Zambia	2.158	2.112	1.176	2.529
	(< 0.001)	(< 0.001)	(0.304)	(< 0.001)
Admin size	0.935	0.991	0.988	0.792
	(0.177)	(0.762)	(0.790)	(0.014)
Constant	0.532	0.223	1.110	0.005
	(0.607)	(0.649)	(0.922)	(0.001)
Observations	34,978	21,495	34,738	34,976
Pseudo R-squared	0.122	0.0971	0.0847	0.171

Adjusted odds ratios (AOR) with *P*-values in parentheses. *P*-values are two-sided and derived from multivariable logistic regression models. Sample sizes (n) for each model are listed in the Observations row. PV: Physical Violence; PV-family: PV from family members; PV-partner: PV from partner; PV-peer: PV from peer; PV-community: PV from adult in the community.

Table 9. Logistic regression results of political violence and past year VAC (split time periods).

	PV	SV	EV	AV	MV
	AOR (*P*-value)	AOR (*P*-value)	AOR (*P*-value)	AOR (*P*-value)	AOR (*P*-value)
Political violence (1-5 years)	0.906 (0.095)	1.009 (0.868)	0.970 (0.530)	0.934 (0.167)	0.939 (0.245)
Political violence (6-15 years)	1.091 (0.064)	1.018 (0.685)	1.072 (0.060)	1.082 (0.040)	1.089 (0.054)
Respondent age	0.747 (0.001)	2.248 (< 0.001)	1.128 (0.381)	0.920 (0.314)	1.360 (0.017)
Respondent age squared	1.004 (0.082)	0.980 (< 0.001)	0.995 (0.245)	1.000 (0.869)	0.990 (0.006)
Gender	0.917 (0.209)	2.234 (< 0.001)	1.021 (0.814)	1.182 (0.014)	1.286 (0.004)
Marital status	0.808 (0.020)	0.425 (< 0.001)	0.618 (< 0.001)	0.605 (< 0.001)	0.568 (< 0.001)
Child in school	1.678 (< 0.001)	1.253 (0.006)	1.154 (0.107)	1.465 (< 0.001)	1.493 (< 0.001)
Respondent worked	1.504 (< 0.001)	1.136 (0.150)	1.580 (< 0.001)	1.492 (< 0.001)	1.442 (< 0.001)
Poorest household	0.989 (0.919)	0.808 (0.071)	0.803 (0.075)	0.930 (0.439)	0.759 (0.039)
Poorer household	0.973 (0.799)	0.789 (0.035)	0.941 (0.636)	0.915 (0.339)	0.850 (0.197)
Average household	1.051 (0.648)	0.868 (0.214)	0.785 (0.054)	0.967 (0.708)	0.846 (0.182)
Richer household	0.973 (0.790)	1.083 (0.421)	0.951 (0.618)	1.006 (0.942)	0.979 (0.861)
Cote d'Ivoire	2.843 (< 0.001)	6.020 (< 0.001)	1.638 (< 0.001)	2.731 (< 0.001)	4.180 (< 0.001)
Kenya	2.619 (< 0.001)	3.662 (< 0.001)	0.861 (0.373)	2.194 (< 0.001)	2.214 (< 0.001)
Malawi	4.013 (< 0.001)	5.116 (< 0.001)	3.248 (< 0.001)	3.932 (< 0.001)	5.327 (< 0.001)
Mozambique	1.939 (0.009)	6.235 (< 0.001)	0.700 (0.064)	2.136 (0.002)	2.043 (0.002)
Namibia	2.601 (< 0.001)	3.757 (< 0.001)	1.401 (0.083)	2.309 (< 0.001)	2.755 (< 0.001)
Nigeria	2.140 (< 0.001)	5.276 (< 0.001)	1.771 (< 0.001)	2.449 (< 0.001)	3.031 (< 0.001)
Uganda	3.562 (< 0.001)	9.256 (< 0.001)	2.228 (< 0.001)	4.312 (< 0.001)	5.127 (< 0.001)
Zambia	1.943 (< 0.001)	2.879 (< 0.001)	1.828 (0.002)	2.014 (< 0.001)	2.297 (< 0.001)
Admin size	0.931 (0.063)	0.881 (0.011)	0.942 (0.200)	0.910 (0.010)	0.918 (0.086)
Constant	4.432 (0.070)	0.000 (< 0.001)	0.042 (0.008)	0.725 (0.669)	0.003 (< 0.001)
Observations	35,325	34,758	34,940	35,418	34,216
Pseudo R-squared	0.0835	0.0612	0.0421	0.0615	0.0559

Adjusted odds ratios (AOR) with *P*-values in parentheses. *P*-values are two-sided and derived from multivariable logistic regression models. Sample sizes (n) for each model are listed in the Observations row. PV: Physical Violence; SV: Sexual Violence; EV: Emotional Violence; AV: Any Violence; MV: Multiple Violence.
