# Supplementary material for: Past political violence and interpersonal violence against children and youth in Africa
Source: Nat Commun. 2026 Apr 13;17:3044. doi: 10.1038/s41467-026-71075-x (PMC13076750; doi:10.1038/s41467-026-71075-x)
Supplement: Supplementary file 2 — Reporting Summary [file 41467_2026_71075_MOESM2_ESM.pdf]

nature portfolio

Corresponding author(s): Tilman Brück

Last updated by author(s): Jan 16, 2026

Reporting Summary

Nature Portfolio wishes to improve the reproducibility of the work that we publish. This form provides structure for consistency and transparency in reporting. For further information on Nature Portfolio policies, see our Editorial Policies and the Editorial Policy Checklist.

Statistics

For all statistical analyses, confirm that the following items are present in the figure legend, table legend, main text, or Methods section.

n/a	Confirmed	
[ ]	[x]	The exact sample size (*n*) for each experimental group/condition, given as a discrete number and unit of measurement
[x]	[ ]	A statement on whether measurements were taken from distinct samples or whether the same sample was measured repeatedly
[ ]	[x]	The statistical test(s) used AND whether they are one- or two-sided<br>*Only common tests should be described solely by name; describe more complex techniques in the Methods section.*
[ ]	[x]	A description of all covariates tested
[ ]	[x]	A description of any assumptions or corrections, such as tests of normality and adjustment for multiple comparisons
[ ]	[x]	A full description of the statistical parameters including central tendency (e.g. means) or other basic estimates (e.g. regression coefficient) AND variation (e.g. standard deviation) or associated estimates of uncertainty (e.g. confidence intervals)
[ ]	[x]	For null hypothesis testing, the test statistic (e.g. *F*, *t*, *r*) with confidence intervals, effect sizes, degrees of freedom and *P* value noted<br>*Give P values as exact values whenever suitable.*
[x]	[ ]	For Bayesian analysis, information on the choice of priors and Markov chain Monte Carlo settings
[x]	[ ]	For hierarchical and complex designs, identification of the appropriate level for tests and full reporting of outcomes
[x]	[ ]	Estimates of effect sizes (e.g. Cohen's *d*, Pearson's *r*), indicating how they were calculated

Our web collection on statistics for biologists contains articles on many of the points above.

Software and code

Policy information about availability of computer code

Data collection	Not applicable as we exclusively used secondary data in our project.
Data analysis	We used Stata 18 in our analysis of the secondary data.

For manuscripts utilizing custom algorithms or software that are central to the research but not yet described in published literature, software must be made available to editors and reviewers. We strongly encourage code deposition in a community repository (e.g. GitHub). See the Nature Portfolio guidelines for submitting code & software for further information.

Data

Policy information about availability of data

All manuscripts must include a data availability statement. This statement should provide the following information, where applicable:

- Accession codes, unique identifiers, or web links for publicly available datasets
- A description of any restrictions on data availability
- For clinical datasets or third party data, please ensure that the statement adheres to our policy

For this secondary data analysis project, the merged scientific use file, including de-identified participant data, underlying the analysis are available on the ReShare web site of the UK Data Service under accession code XXX [add hyperlink and DOI here].

Research involving human participants, their data, or biological material

Policy information about studies with human participants or human data. See also policy information about sex, gender (identity/presentation), and sexual orientation and race, ethnicity and racism.

Reporting on sex and gender	We use secondary data from the Violence Against Children and Youth Surveys (VACS). A central concern of the VACS project is to understand how girls and boys, women and men experience violence differently. Likewise our research in interested in these differentiated outcomes. Hence the data we obtained is gender-differentiated, according to the guidelines by the VACS project. Our study also reports outcomes according to these classifications.
Reporting on race, ethnicity, or other socially relevant groupings	Our study did not include as a topic either race or ethnicity.
Population characteristics	We did not collect any data ourselves. The survey data we used derive from nationally representative household surveys focusing on adolescents and young people aged 13-24 years.
Recruitment	Not applicable.
Ethics oversight	As a secondary data analysis, this study did not require approval by an ethics committee.

Note that full information on the approval of the study protocol must also be provided in the manuscript.

Field-specific reporting

Please select the one below that is the best fit for your research. If you are not sure, read the appropriate sections before making your selection.

☐ Life sciences ☒ Behavioural & social sciences ☐ Ecological, evolutionary & environmental sciences

For a reference copy of the document with all sections, see nature.com/documents/nr-reporting-summary-flat.pdf

Behavioural & social sciences study design

All studies must disclose on these points even when the disclosure is negative.

Study description	Quantitative cross-sectional survey data merged with violent conflict event data.
Research sample	Nationally representative household surveys focusing on adolescents and young people aged 13-24 years from Côte d'Ivoire, Kenya, Malawi, Mozambique, Namibia, Nigeria, Uganda, Zambia, and Zimbabwe, constituting all available VACS data at the time of analysis. Our sample includes 15,888 adolescents aged 13-17 years and 19,548 young adults aged 18-24 years. Female respondents are 66.7% of the sample due to an oversampling of girls. The average respondent was 18 years old; 49% were attending school at the time of the survey and 24% had worked during the past 12 months.
Sampling strategy	We included all VACS datasets that were made available to us at the time of our analysis. Each survey adopts a three-stage cluster split-sample design that selects one respondent per household and assigns male and female interviews to different enumeration areas, ensuring boys and girls are never interviewed in the same geographical areas. As our project is a secondary data analysis, we refer to the reference describing the sampling strategy and the subsequent questions of the underlying VACS data: Centers for Disease Control and Prevention. Violence Against Children and Youth Surveys. https://www.cdc.gov/violence-against-children/about/index.html (2024).
Data collection	The VACS were collected by the various national VACS survey teams between September 2013 (in Malawi) and July 2019 (in Mozambique), as described in the Supplementary File. We were not involved in the data collection. Our research project started after the data collection was completed; respondents hence could not have been aware of our study hypothesis.
Timing	The surveys were collected as follows: Côte d'Ivoire: June 2018, Kenya: January 2019, Malawi: September 2013, Mozambique: July 2019, Namibia: March 2019, Nigeria: May 2014, Uganda: September 2015, Zambia: August 2014, and Zimbabwe: January 2017.
Data exclusions	No data were excluded from the analysis.
Non-participation	We do not know if there was non-participation in the original VACS data collection. We used the full available samples.
Randomization	The VACS data collection did not involve any randomization.

Reporting for specific materials, systems and methods

We require information from authors about some types of materials, experimental systems and methods used in many studies. Here, indicate whether each material, system or method listed is relevant to your study. If you are not sure if a list item applies to your research, read the appropriate section before selecting a response.

Materials & experimental systems

n/a	Involved in the study
☒	☐ Antibodies
☒	☐ Eukaryotic cell lines
☒	☐ Palaeontology and archaeology
☒	☐ Animals and other organisms
☒	☐ Clinical data
☒	☐ Dual use research of concern
☒	☐ Plants

Methods

n/a	Involved in the study
☒	☐ ChIP-seq
☒	☐ Flow cytometry
☒	☐ MRI-based neuroimaging

Plants

Seed stocks	n.a.
Novel plant genotypes	n.a.
Authentication	n.a.
